# 3-Chloro­azepan-2-one

**DOI:** 10.1107/S1600536810002060

**Published:** 2010-01-23

**Authors:** De-Cai Wang, Dong-Mei Fan, Hua-Quan Liu, Ping-Kai Ou-yang

**Affiliations:** a State Key Laboratory of Materials-Oriented Chemcial Engineering, College of Life Science and Pharmaceutical Engineering, Nanjing University of Technology, Xinmofan Road No. 5 Nanjing, Nanjing 210009, People’s Republic of China

## Abstract

In the title compound, C_6_H_10_ClNO, an inter­mediate for the production of lysine, there are intra­molecular C—H⋯Cl hydrogen bonds.

## Related literature

For the preparation of the title compound, see: Wineman *et al.* (1958 ▶). For puckering parameters, see: Cremer & Pople (1975 ▶). For bond-length data, see: Allen *et al.* (1987 ▶).
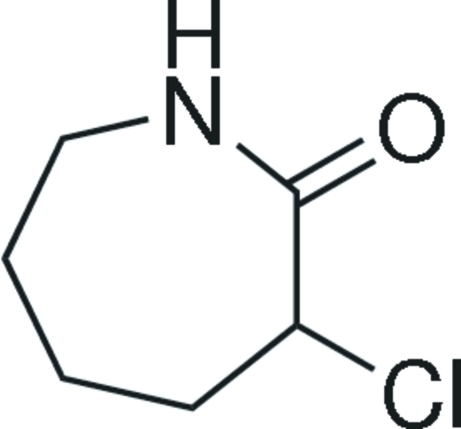


## Experimental

### 

#### Crystal data

C_6_H_10_ClNO
*M* *_r_* = 147.60
Monoclinic, 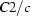

*a* = 18.776 (4) Å
*b* = 7.3440 (15) Å
*c* = 11.109 (2) Å
β = 103.65 (3)°
*V* = 1488.6 (5) Å^3^
*Z* = 8
Mo *K*α radiation
μ = 0.43 mm^−1^
*T* = 293 K
0.30 × 0.20 × 0.20 mm

#### Data collection

Enraf–Nonius CAD-4 diffractometer
Absorption correction: ψ scan (North *et al.*, 1968 ▶) *T*_min_ = 0.881, *T*_max_ = 0.918
2654 measured reflections
1345 independent reflections
1107 reflections with *I* > 2σ(*I*)
*R*_int_ = 0.020
3 standard reflections every 200 reflections  intensity decay: 1%

#### Refinement

*R*[*F* ^2^ > 2σ(*F* ^2^)] = 0.044
*wR*(*F* ^2^) = 0.148
*S* = 1.01
1345 reflections
82 parameters
H-atom parameters constrained
Δρ_max_ = 0.36 e Å^−3^
Δρ_min_ = −0.33 e Å^−3^

### 

Data collection: *CAD-4 Software* (Enraf–Nonius, 1989 ▶); cell refinement: *CAD-4 Software*; data reduction: *XCAD4* (Harms & Wocadlo, 1995 ▶); program(s) used to solve structure: *SHELXS97* (Sheldrick, 2008 ▶); program(s) used to refine structure: *SHELXL97* (Sheldrick, 2008 ▶); molecular graphics: *SHELXTL* (Sheldrick, 2008 ▶); software used to prepare material for publication: *PLATON* (Spek, 2009 ▶).

## Supplementary Material

Crystal structure: contains datablocks global, I. DOI: 10.1107/S1600536810002060/gw2071sup1.cif

Structure factors: contains datablocks I. DOI: 10.1107/S1600536810002060/gw2071Isup2.hkl

Additional supplementary materials:  crystallographic information; 3D view; checkCIF report

## Figures and Tables

**Table 1:** Hydrogen-bond geometry (Å, °)

*D*—H⋯*A*	*D*—H	H⋯*A*	*D*⋯*A*	*D*—H⋯*A*
C1—H1*B*⋯Cl	0.97	2.82	3.215 (3)	105
C3—H3*B*⋯Cl	0.97	2.80	3.374 (3)	119

**Fig. 1.**
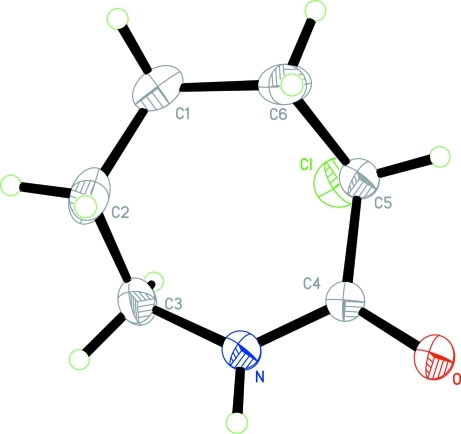
The molecular structure of (I), showing the atom-numbering scheme and displacement ellipsoids at the 30% probability level.

**Fig. 2.**
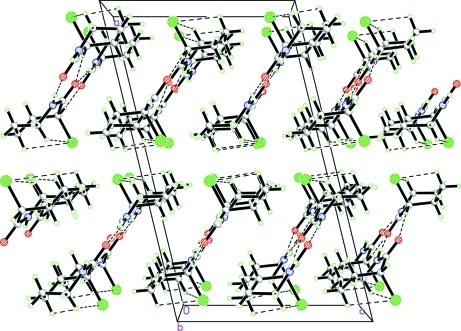
A packing diagram of (I). The intermolecular hydrogen bonds are shown as dashed lines.

## supplementary crystallographic information

### Comment

Some derivatives of 3-chloroazepan-2-one is important chemical material. We report here the crystal structure of the title compound, (I). The molecular structure of (I) is shown in Fig. 1, and the selected geometric parameters are given in Table 1. The bond lengths and angles (Table 1) are within normal ranges (Allen *et al.*, 1987). The seven-membered ring A (N/C1-C6) is not planar, having total puckering amplitude, Q_T_, of 0.702 (2) Å (Cremer & Pople, 1975).

The molecular structure of (I) is shown in Fig. 1. A packing diagram of (I) is shown in Fig. 2, where the dash line indicates C—H···Cl hydrogen bond.

### Experimental

18.2 g (100 mmol) 3,3-dichloro-2-oxohexamethyleneimine, 2 g. 5% palladium-on-charcoal and 18 g. (220 mmol) sodium acetate were added into 100 ml glacial acetic acid. The mixture was placed in in a shaker under hydrogen (2 atm. initial pressure) until one equivalent of hydrogen was absorbed. The catalyst and sodium chloride were removed by filtration. The filtrate was neutralized and extracted with chloroform and concentrated, and then recrystallized by n-hexane to give 18.1g white solid (87.4%). (Wineman *et al.*, 1958) Pure compound (I) was obstained by crystallizing from acetic acid. Crystals of (I) suitable for X-ray diffraction were obstained by slow evaporation of an ethanol solution.

### Refinement

All H atoms bonded to the C atoms were placed geometrically at the distances of 0.93–0.97 Å, and included in the refinement in riding motion approximation with *U*_iso_(H) = 1.2 or 1.5*U*_eq_ of the carrier atom.

### Crystal data

**Table:**

C_6_H_10_ClNO	*F*(000) = 624
*M**_r_* = 147.60	*D*_x_ = 1.317 Mg m^−^^3^
Monoclinic, *C*2/*c*	Mo *K*α radiation, λ = 0.71073 Å
Hall symbol: -C 2yc	Cell parameters from 25 reflections
*a* = 18.776 (4) Å	θ = 9–14°
*b* = 7.3440 (15) Å	µ = 0.43 mm^−^^1^
*c* = 11.109 (2) Å	*T* = 293 K
β = 103.65 (3)°	Block, colourless
*V* = 1488.6 (5) Å^3^	0.30 × 0.20 × 0.20 mm
*Z* = 8	

### Data collection

**Table:**

Enraf–Nonius CAD-4 diffractometer	1107 reflections with *I* > 2σ(*I*)
Radiation source: fine-focus sealed tube	*R*_int_ = 0.020
graphite	θ_max_ = 25.3°, θ_min_ = 2.2°
ω/2θ scans	*h* = 0→22
Absorption correction: ψ scan (North *et al.*, 1968)	*k* = −8→8
*T*_min_ = 0.881, *T*_max_ = 0.918	*l* = −13→12
2654 measured reflections	3 standard reflections every 200 reflections
1345 independent reflections	intensity decay: 1%

### Refinement

**Table:**

Refinement on *F*^2^	Primary atom site location: structure-invariant direct methods
Least-squares matrix: full	Secondary atom site location: difference Fourier map
*R*[*F*^2^ > 2σ(*F*^2^)] = 0.044	Hydrogen site location: inferred from neighbouring sites
*wR*(*F*^2^) = 0.148	H-atom parameters constrained
*S* = 1.01	*w* = 1/[σ^2^(*F*_o_^2^) + (0.1*P*)^2^ + 0.650*P*] where *P* = (*F*_o_^2^ + 2*F*_c_^2^)/3
1345 reflections	(Δ/σ)_max_ < 0.001
82 parameters	Δρ_max_ = 0.36 e Å^−^^3^
0 restraints	Δρ_min_ = −0.33 e Å^−^^3^

### Special details

**Table:**

Geometry. All e.s.d.'s (except the e.s.d. in the dihedral angle between two l.s. planes) are estimated using the full covariance matrix. The cell e.s.d.'s are taken into account individually in the estimation of e.s.d.'s in distances, angles and torsion angles; correlations between e.s.d.'s in cell parameters are only used when they are defined by crystal symmetry. An approximate (isotropic) treatment of cell e.s.d.'s is used for estimating e.s.d.'s involving l.s. planes.
Refinement. Refinement of *F*^2^ against ALL reflections. The weighted *R*-factor *wR* and goodness of fit *S* are based on *F*^2^, conventional *R*-factors *R* are based on *F*, with *F* set to zero for negative *F*^2^. The threshold expression of *F*^2^ > σ(*F*^2^) is used only for calculating *R*-factors(gt) *etc*. and is not relevant to the choice of reflections for refinement. *R*-factors based on *F*^2^ are statistically about twice as large as those based on *F*, and *R*- factors based on ALL data will be even larger.

### Fractional atomic coordinates and isotropic or equivalent isotropic displacement parameters (Å^2^)

**Table:**

	*x*	*y*	*z*	*U*_iso_*/*U*_eq_	
Cl	0.05987 (4)	0.14400 (11)	0.65254 (6)	0.0703 (3)	
O	0.24623 (10)	0.2773 (2)	0.75479 (18)	0.0609 (5)	
N	0.17420 (10)	0.4720 (2)	0.62946 (16)	0.0436 (5)	
H0A	0.2035	0.5567	0.6645	0.052*	
C1	0.08706 (17)	0.2590 (4)	0.3877 (2)	0.0647 (8)	
H1A	0.0840	0.2264	0.3020	0.078*	
H1B	0.0383	0.2471	0.4022	0.078*	
C2	0.11018 (17)	0.4574 (4)	0.4058 (2)	0.0671 (8)	
H2A	0.1587	0.4702	0.3906	0.081*	
H2B	0.0768	0.5305	0.3446	0.081*	
C3	0.11132 (14)	0.5312 (3)	0.5331 (2)	0.0552 (7)	
H3A	0.1113	0.6632	0.5295	0.066*	
H3B	0.0668	0.4936	0.5560	0.066*	
C4	0.19194 (12)	0.3048 (3)	0.66976 (19)	0.0402 (5)	
C5	0.14703 (13)	0.1426 (3)	0.6099 (2)	0.0458 (6)	
H5A	0.1731	0.0334	0.6475	0.055*	
C6	0.13727 (15)	0.1235 (4)	0.4704 (2)	0.0579 (7)	
H6B	0.1853	0.1320	0.4525	0.069*	
H6A	0.1188	0.0021	0.4469	0.069*	

### Atomic displacement parameters (Å^2^)

**Table:**

	*U*^11^	*U*^22^	*U*^33^	*U*^12^	*U*^13^	*U*^23^
Cl	0.0684 (5)	0.0767 (6)	0.0696 (5)	−0.0248 (4)	0.0237 (4)	0.0048 (3)
O	0.0615 (10)	0.0416 (9)	0.0627 (10)	0.0047 (8)	−0.0190 (9)	0.0013 (8)
N	0.0462 (10)	0.0336 (10)	0.0451 (10)	−0.0026 (8)	−0.0014 (8)	0.0018 (8)
C1	0.0739 (18)	0.0727 (18)	0.0402 (13)	−0.0032 (15)	−0.0014 (12)	−0.0069 (12)
C2	0.0781 (19)	0.0712 (18)	0.0448 (14)	−0.0010 (15)	−0.0001 (13)	0.0173 (12)
C3	0.0559 (14)	0.0410 (13)	0.0606 (15)	0.0059 (11)	−0.0024 (12)	0.0090 (11)
C4	0.0426 (11)	0.0364 (11)	0.0389 (11)	0.0013 (9)	0.0040 (9)	0.0006 (9)
C5	0.0488 (12)	0.0370 (11)	0.0469 (12)	−0.0012 (10)	0.0022 (10)	0.0003 (10)
C6	0.0648 (16)	0.0545 (15)	0.0516 (14)	−0.0012 (12)	0.0084 (12)	−0.0158 (11)

### Geometric parameters (Å, °)

**Table:**

Cl—C5	1.808 (3)	C2—H2A	0.9700
O—C4	1.232 (3)	C2—H2B	0.9700
N—C4	1.322 (3)	C3—H3A	0.9700
N—C3	1.460 (3)	C3—H3B	0.9700
N—H0A	0.8600	C4—C5	1.519 (3)
C1—C2	1.520 (4)	C5—C6	1.524 (3)
C1—C6	1.521 (4)	C5—H5A	0.9800
C1—H1A	0.9700	C6—H6B	0.9700
C1—H1B	0.9700	C6—H6A	0.9700
C2—C3	1.510 (4)		
			
C4—N—C3	128.35 (19)	N—C3—H3B	108.7
C4—N—H0A	115.8	C2—C3—H3B	108.7
C3—N—H0A	115.8	H3A—C3—H3B	107.6
C2—C1—C6	115.5 (2)	O—C4—N	120.6 (2)
C2—C1—H1A	108.4	O—C4—C5	118.68 (19)
C6—C1—H1A	108.4	N—C4—C5	120.71 (18)
C2—C1—H1B	108.4	C4—C5—C6	116.0 (2)
C6—C1—H1B	108.4	C4—C5—Cl	108.92 (15)
H1A—C1—H1B	107.5	C6—C5—Cl	111.58 (17)
C3—C2—C1	114.1 (2)	C4—C5—H5A	106.6
C3—C2—H2A	108.7	C6—C5—H5A	106.6
C1—C2—H2A	108.7	Cl—C5—H5A	106.6
C3—C2—H2B	108.7	C1—C6—C5	117.6 (2)
C1—C2—H2B	108.7	C1—C6—H6B	107.9
H2A—C2—H2B	107.6	C5—C6—H6B	107.9
N—C3—C2	114.2 (2)	C1—C6—H6A	107.9
N—C3—H3A	108.7	C5—C6—H6A	107.9
C2—C3—H3A	108.7	H6B—C6—H6A	107.2
			
C6—C1—C2—C3	−62.8 (3)	N—C4—C5—C6	55.0 (3)
C4—N—C3—C2	−63.1 (3)	O—C4—C5—Cl	109.8 (2)
C1—C2—C3—N	76.1 (3)	N—C4—C5—Cl	−71.8 (2)
C3—N—C4—O	−178.0 (2)	C2—C1—C6—C5	61.3 (4)
C3—N—C4—C5	3.6 (4)	C4—C5—C6—C1	−71.6 (3)
O—C4—C5—C6	−123.4 (2)	Cl—C5—C6—C1	53.9 (3)

### Hydrogen-bond geometry (Å, °)

**Table:**

*D*—H···*A*	*D*—H	H···*A*	*D*···*A*	*D*—H···*A*
C1—H1B···Cl	0.97	2.82	3.215 (3)	105
C3—H3B···Cl	0.97	2.80	3.374 (3)	119
